# C-Abl Inhibitor Imatinib Enhances Insulin Production by β Cells: C-Abl Negatively Regulates Insulin Production via Interfering with the Expression of NKx2.2 and GLUT-2

**DOI:** 10.1371/journal.pone.0097694

**Published:** 2014-05-16

**Authors:** Chang-Qing Xia, Pengcheng Zhang, Shiwu Li, Lihui Yuan, Tina Xia, Chao Xie, Michael J. Clare-Salzler

**Affiliations:** 1 Department of Hematology, Xuanwu Hospital, Capital Medical University, Bejing, China; 2 Department of Pathology, Immunology and Laboratory Medicine, Diabetes Center of Excellence, University of Florida, Gainesville, Florida, United States of America; University of Catanzaro Magna Graecia, Italy

## Abstract

Chronic myelogenous leukemia patients treated with tyrosine kinase inhibitor, Imatinib, were shown to have increased serum levels of C-peptide. Imatinib specifically inhibits the tyrosine kinase, c-Abl. However, the mechanism of how Imatinib treatment can lead to increased insulin level is unclear. Specifically, there is little investigation into whether Imatinib directly affects β cells to promote insulin production. In this study, we showed that Imatinib significantly induced insulin expression in both glucose-stimulated and resting β cells. In line with this finding, c-Abl knockdown by siRNA and overexpression of c-Abl markedly enhanced and inhibited insulin expression in β cells, respectively. Unexpectedly, high concentrations of glucose significantly induced c-Abl expression, suggesting c-Abl may play a role in balancing insulin production during glucose stimulation. Further studies demonstrated that c-Abl inhibition did not affect the major insulin gene transcription factor, pancreatic and duodenal homeobox-1 (PDX-1) expression. Of interest, inhibition of c-Abl enhanced NKx2.2 and overexpression of c-Abl in β cells markedly down-regulated NKx2.2, which is a positive regulator for insulin gene expression. Additionally, we found that c-Abl inhibition significantly enhanced the expression of glucose transporter GLUT2 on β cells. Our study demonstrates a previously unrecognized mechanism that controls insulin expression through c-Abl-regulated NKx2.2 and GLUT2. Therapeutic targeting β cell c-Abl could be employed in the treatment of diabetes or β cell tumor, insulinoma.

## Introduction

Imatinib Mesylate, commonly known as Gleevec, is a selective tyrosine kinase inhibitor which targets the Abelson tyrosine kinase, also known as Abl1 and c-Abl, platelet derived growth factor receptor (PDGFR), transmembrane receptor tyrosine kinase, and ABL-related genes [1], [2]. In recent years, Imatinib has been a marketed effective drug for chronic myelogenous leukemia (CML) and gastrointestinal stromal tumors (GIST) [3], and the two listed disorders are caused by BCR-ABL and c-kit oncogenes [4], respectively. Interestingly, recent studies have implicated Imatinib in ameliorating symptoms of diabetes, a disorder with a completely different pathogenesis from CML and GIST. For a number of patients who have both type 2 diabetes and CML, Imatinib has improved symptoms for both disorders [5]. This finding prompts investigations into the mechanism of action of the Imatinib drug at the cellular level, and whether it is also therapeutic for type 1 diabetes. Type 1 diabetes, also known as insulin-dependent diabetes is a chronic autoimmune disorder affecting approximately 1/300 persons in the United State. It is caused by genetic and/or environmental factors interacting to induce an autoimmune response that destroys the islet β cells within the pancreas. This process involves autoreactive CD4^+^ and CD8^+^ T cells, B lymphocytes, and activation of the innate immune system [6]. Insulin-dependent (type 1) diabetes ensues when this autoimmune attack coordinated with a pro-inflammatory environment results in the death of β cells to the extent that the residual islet β cells are unable to produce adequate insulin to regulate blood glucose levels in a normal range.

When Imatinib was orally administered to nonobese diabetic mice (NOD), a typical human type 1 diabetes mouse model, the drug dramatically prevented NOD mice from developing type 1 diabetes [7]. The most striking finding in this report was that Imatinib rapidly reversed diabetes in NOD mice with new onset diabetes showing that around 50% of the diabetic mice became euglycemia within a week of drug administration, and almost all diabetic mice were reversed within 10 days. While the authors attributed this effect to the possible anti-inflammatory activities of Imatinib to protect the residual β cells, the pancreatic histological study results failed to support this idea because there was no difference in terms of insulitic lesions between the treated and untreated groups [7]. Also, it is an unwarranted conclusion that anti-inflammation leads to diabetes reversal under the situation that diabetes recurs within a week in the majority of the mice upon the discontinuation of Imatinib which has already been administered for three weeks. Imatinib’s rapid diabetes-reversing effect is also difficult to be explained by its anti-apoptotic effect on β cells demonstrated by a previous study [8] because in diabetic NOD mice the residual β cells are not able to maintain euglycemia even if they are no longer undergoing apoptosis unless the ability of the residual β cells to secrete insulin is enhanced simultaneously. We believe the mechanism underlying Immatinib’s diabetes-reversing effect is still unclear. Based on the findings that Imatinib can quickly reverse new onset diabetes [7], two possibilities likely exist: one is that Imatinib enhances insulin production by the limited number of residual β cells in new-onset diabetic mice; the other is that Imatinib improves the sensitivity of peripheral tissues to insulin so that insufficient insulin would still be able to control blood glucose. Although the latter is supported by the previous study showing that Imatinib improves insulin sensitivity and glucose disposal rates in rats fed a high-fat diet [9], a recent study shows the contradictory, demonstrating that c-Abl activation, and not inhibition, enhances insulin sensitivity in liver, muscle and fat cells [10], the major tissues responding to insulin in our body. Therefore, it is questionable whether Imatinib truly improves insulin sensitivity. We think the most plausible explanation would be that Imatinib enhances insulin production by the residual β cells. In this study, we hypothesize that tyrosine kinase c-Abl negatively regulates insulin expression in β cells, therefore, inhibition of c-Abl enhances insulin production by β cells.

To test this hypothesis, in this study, we investigated how c-Abl inhibition using different approaches affected the insulin production in β cells. Our results indeed demonstrated that Imatinib up-regulated insulin expression not only in glucose-stimulated β cells but surprisingly in non-stimulated resting β cells. SiRNA interference of the c-Abl gene markedly enhanced insulin production. Further analysis demonstrated that c-Abl tyrosine kinase affected insulin gene expression without involvement of PDX-1, which is known to be the major transcription factor regulating insulin gene expression. However, overexpression of c-Abl in β cells led to down-regulation of insulin gene expression accompanied by the reduction of NKx2.2, a positive regulator of insulin gene expression. Imatinib treatment of β cells leads to increased levels of NKx2.2. Intriguingly, we also unraveled that c-Abl inhibition markedly promoted the expression of glucose transporter, GLUT2, on β cells. To our knowledge, this is the first report on the role of C-Abl in inhibiting β cell insulin expression possibly via negatively regulating NKx2.2 and GLUT2.

## Materials and Methods

### Mouse Insulin and C-peptide ELISA Experimentation

Mouse pancreatic β cell line NIT-1 (derived from NOD β cells, originally purchased from ATCC and maintained in our laboratory) were cultured in 1 ml of DMEM media with 4.5 mM glucose and supplemented with fetal bovine serum (FBS), HEPES, MEM non-essential amino acids, and penicillin/streptomycin. The numbers of cells utilized in the various experiments were different but kept consistent in the same experiment. The cells were cultured for various times as indicated in the specific experiments under different conditions: media only, high concentration of glucose, imatinib, glucose and imatinib. Supernatant in some experiments was collected from each culture condition and measured for insulin and/or C-peptide with mouse ultrasensitive ELISA kits (ALPCO Diagnostics, Salem, NH). The assay was analyzed using spectrometer (BioTek Instruments, Inc., Winooski, VT) at 450 nm and 630 nm and insulin concentration was calculated according to instructions from the manufacturer.

### RT-PCR Experimentation

NIT-1 cells were cultured in 1 ml of DMEM media with low glucose (4.5 mM) supplemented with FBS, HEPES, MEM non-essential amino acids, and penicillin/streptomycin. The cells were cultured for 6 hrs under different culture conditions as indicated in each experiment. After cell culture, mRNA was extracted from conditioned NIT-1 cells using Qiagen RNeasy Mini Kit (Qiagen Inc., Valencia, CA). The mRNA samples were reverse transcribed and evaluated via real-time RT-PCR. For insulin, pdx-1 and neuro D1 real-time PCR, samples were processed with optimized SYBR green protocol (Sigma-Aldrich, St. Louis, MO), and data was analyzed using Opticon MJ software (Promega Biosciences, Inc., San Luis Obispo, CA). Alternatively, for c-Abl real-time PCR, the cDNA samples were processed and evaluated following the TaqMan protocol, using hydrolysis probes for detection (Roche Ltd., Basel, Switzerland).

### C-Abl Cytoimmunological Staining in NIT-1 Cells

NIT-1 cells were spun to the slides by cytospinning. The cells in the slides were fixed with 4% paraformaldehyde for 5 minutes and then blocked for 10 minutes at room temperature with 10% normal goat serum. The cells were spun onto the glides and stained with c-abl antibody for 1 hour at room temperature (1∶200, Cell Signaling Technology) followed by secondary antibody, goat anti rabbit AF555 for 1 hour at room temperature (1∶1000, Invitrogen Technology). All the washing steps were used with 1X TBS solution. Imaging was done using Zeiss Axioskop microscope.

### Mouse c-Abl siRNA Transfection on NIT-1 Cells

NIT-1 cells were cultured in low glucose DMEM media as mentioned above. The c-Abl or control siRNA was incubated with NIT-1 cells following the protocol from the siRNA transfection kit (Santa Cruz Biotech, Inc., Santa Cruz, CA). Thereafter, the siRNA transfected NIT-1 cells were incubated for 6 hours in the presence of 16 mM glucose. Insulin gene expression in NIT-1 cells transfected with control siRNA and cultured in low glucose DMEM media served as an additional control. The supernatants were collected from the cultures and assayed for insulin expression by ELISA as aforementioned. Additionally, mRNA was extracted from c-Abl or control siRNA transfected NIT-1 cells, and c-Abl and insulin gene expression was evaluated using real-time RT-PCR as described above.

### Luciferase Assay

In a set of experiments, cells of the 293 cell line (provided by Dr. Lijun Yang at University of Florida) were co-transfected with mouse Pdx-1 promoter-driven firefly luciferase reporter gene along with control plasmid (pCDNA3), *c-Abl* plasmid or *pdx-1* plasmid (self-regulated gene). Twenty-four hrs later, the luciferase activities in the above conditioned cells were measured using the Dual Luciferase Reporter Kit (Promega). To evaluate the gene specific changes in expression, firefly luciferase activity was normalized to that of *Renilla* luciferase, included in kit.

### Overexpression of c-Abl in β Cells by Retrovirus-*c-Abl* Transfection and Examination of Insulin, PDX-1, NKx6.1 and NKx2.2 Gene Expression

The retrovirus-c-Abl and lentivirus-GFP plasmids were prepared according the method previously reported [11]. Then, NIT-1 cells (β cell line) were transdued with retrovirus carrying the *c-Abl* gene (2×10^6^ transducing units (TU)/ml) or control vector (lentivirus-GFP) (2×10^6^ TU/ml). Forty-eight hours later, the transduced β cells were examined by real-time PCR for the expression of insulin-I and II, PDX-1, NKx2.2, and NKx6.1. The data were normalized to β actin and fold-change was calculated relative to NIT-1 cells transfected with Lentivirus GFP, validated using Opticon MJ software.

### Western blot Assay for NKx2.2 and PDX-1 Expression in NIT-1 Cells

NIT-1 cells were cultured in low glucose DMEM or in DMEM with glucose 16 mM in the presence of Imatinib for 24 hrs. Thereafter, the cells were harvested and processed using Western blotting lysis buffer (Qiagen). The cell lysates were used to run SDS-PAGE gel (10%). The proteins were transferred to a nitrocellulose blotting membrane, which was then used for blotting by anti-NKx2.2 and PDX-1 antibodies (rabbit polyclonal antibody, Abcam, Cambridge, MA), respectively at a dilution of 1∶500. Horseradish peroxidase (HRP)-conjugated goat anti-rabbit secondary antibody was used at 1∶2000 diluation. The procedure of western blot was performed following the instruction from the manufacturer (Qiagen). The densitometric analysis of the target proteins relative to the level of β-actin or GAPDH was performed using a densitometry analysis program (ImageJ 1.47, NIH).

### Measurement of GLUT2 Transcription Levels Using Real-time RT-PCR

NIT-1 cells were cultured in low glucose DMEM with or without 3 µM Imatinib for 6 hrs. Then the cells in each condition were harvested and the RNA was extracted using Qiagen RNeasy Mini Kit (Qiagen Inc., Valencia, CA). Real-time RT-PCR for GLUT2 gene expression was performed following the instruction from the manufacturer (Qiagen). The expression level of GLUT2 in Imatinib-treated NIT-1 cells was calculated relative to the level of GLUT2 in NIT-1 cells cultured in the absence of Imatinib.

### Western Blotting for GLUT2 Expressed on β Cells

NIT-1 cells were cultured in low glucose DMEM alone or with 8 mM glucose, 16 mM glucose, 3 µM Imatinib, 8 mM glucose and 3 µM Imatinib, or 16 mM glucose and 3 µM Imatinib for 24 hrs. Thereafter, the cells in each culture condition were harvested and processed using Western blotting lysis buffer (Qiagen). The cell lysates were used to run SDS-PAGE gel (7.5%). The proteins on the gel were transferred to a nitrocellulose blotting membrane, which was then used for blotting by anti-GLUT2 antibodies (goat polyclonal antibody, LifeSpan BioScience) at a dilution of 1∶500. Rabbit anti-goat secondary antibody was used at 1∶2000 diluation. The densitometry of each GLUT2 band relative to the level of β-actin band of the corresponding sample was performed using a densitometry analysis program (ImageJ 1.47, NIH ).

### Statistical Analysis

The unpaired student *t* test was used for comparison of two independent samples. For the experiments with multiple groups, we employed one-way ANOVA with post hoc test, or two-way ANOVA followed by a Bonferroni post-test. The difference with p<0.05 was considered to be significant.

## Results

### Inhibition of Tyrosine Kinase c-Abl Synergizes with Glucose Stimulation in Inducing Insulin Expression

**Figure 1:**
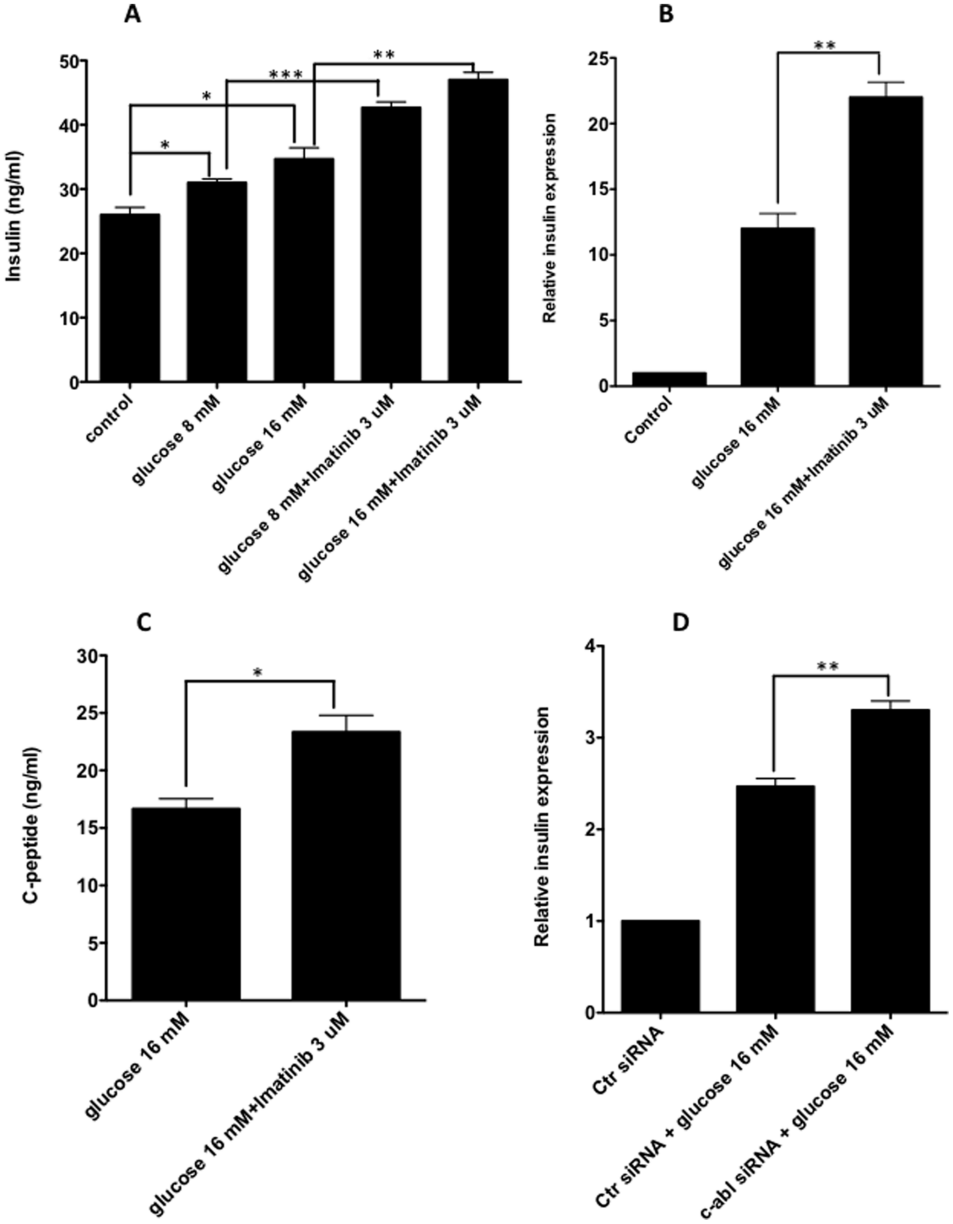
Inhibition of c-Abl significantly enhance glucose-stimulated insulin production by β cells. **A**. NIT-1 cells were cultured in the low glucose media (glucose 4.5 mM) with glucose 8 mM, 16 mM, 8 mM plus Imatinib 3 µM or 16 mM plus Imatinib 3 µM for 6 hrs. The insulin concentration was measured using ELISA. The similar results were obtained in 3 independent experiments. Two-way ANOVA with Bonferroni post-test was performed. **B**. NIT-1 cells were cultured in the low glucose media (glucose 4.5 mM) with glucose 16 mM or 16 mM plus Imatinib 3 µM for 6 hrs, insulin gene expression was examined by real-time RT-PCR. The levels of insulin gene expression were normalized relative to β actin. The results were reproduced in 3 independent experiments. Student t test was performed. **C**. In the cultures of **B** above, C-peptide concentration in each incuation was examined using ELISA. Student t test was performed. **D**. NIT-1 cells were transfected with c-Abl siRNA or control siRNA for 24 hrs, then were stimulated with 16 mM glucose for 6 hrs. Insulin gene expression was examined by real-time RT-PCR, the data were calculated relative to the group with NIT-1 cells transfected with control siRNA without glucose stimulation. Three independent experiments were performed with similar results. Student t test was performed. *: p<0.05, **:p<0.01,***:p<0.001.

Previous evidence showed that administration of the c-Abl tyrosine kinase inhibitor, Imatinib, reverses diabetes in new onset diabetic NOD mice [7], and improves type 2 diabetes [5]. Although this diabetes-reversal effect of Imatinib has been suggested to be associated with its anti-inflammatory effect [7] as well as improving insulin sensitivity [9], whether Imatinib has direct effects on β cells to promote insulin production is yet to be addressed. If β cells were shown to increase insulin levels, then Imatinib can be a candidate therapy for diabetes including type 1 and type 2 diabetes. To determine the role of Imatinib in regulating insulin expression, we treated mouse β cell line, NIT-1 cells, with high concentrations of glucose and with or without Imatinib to assess insulin expression. As expected, glucose significantly promoted insulin expression at both the protein and mRNA levels (Fig. 1A and B). Surprisingly, we discovered that Imatinib treatment along with glucose stimulation led to further increase in insulin expression by the β cells (Fig. 1A and B). In line with these results, independent experiments showed that Imatinib enhanced the capacity of β cells to secrete C-peptide, a more stable form derived from pro-insulin (Fig. 1C). Because Imatinib is a specific c-Abl inhibitor, the results suggest that c-Abl is a negative regulator for glucose-induced insulin production. To further validate the role of c-Abl in β cell insulin expression, we knocked down the *c-Abl* gene by transfection of *c-Abl* siRNA in NIT-1 β cells to study the influence of c-Abl on insulin production induced by glucose. In corroboration with the above findings, the knockdown of *c-Abl* significantly enhanced glucose-induced insulin production (Fig. 1D).

### C-Abl Plays an Important Role in Controlling Insulin Production in Resting β Cells

**Figure 2:**
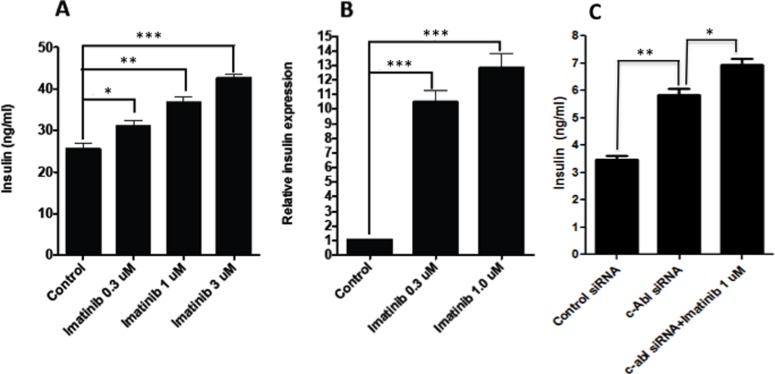
Inhibition of c-Abl promotes insulin production by β cells in resting state. NIT-1 cells were cultured in low glucose DMEM medium (glucose 4.5 mM) for a few days. **A**. The NIT-1 cells were harvested and then cultured in low glucose DMEM medium with different conditions: Imatinib 0 µM, 0.3 µM, 1 µM or 3 µM for 6 hrs. The supernatants from all cultures were harvested and measured for insulin production by ELISA. The results presented were from a representative of three independent experiments. Two-way ANOVA with Bonferroni post-test was performed. **B**. The NIT-1 cells were cultured in low glucose DMEM medium with Imatinib 0 µM, 0.3 µM or 1 µM for 6 hrs. The insulin gene expression was examined by real-time RT-PCR. The levels of insulin gene expression were normalized relative to β actin. The experiments were repeated 3 times with reproducible results. Two-way ANOVA with Bonferroni post-test was performed. **C**. NIT-1 cells were transfected with c-Abl siRNA or control siRNA for 24 hrs. Then the transfected cells were cultured in low glucose DMEM medium for 6 hrs. An additional group was also included by culturing c-Abl siRNA transfected NIT-1 cells with 1 µM Imatinib. The supernatants were harvested from the above cultures and the insulin concentration was examined by ELISA. One-way ANOVA with post hoc test was performed. Similar results were obtained from at least 3 independent experiments. *: p<0.05, **:p<0.01,***:p<0.001.

It is known that β cell produces little insulin when insulin is not needed, which is when the β cells are exposed to the low levels of glucose. However, the mechanisms regulating insulin expression in the resting β cells are not well investigated. To determine whether c-Abl also plays a role in regulating insulin expression in resting β cells, we assessed the effect of c-Abl suppression on insulin production in resting β cells. Intriguingly, we found that inhibition of c-Abl by Imatinib dramatically enhanced insulin production of NIT-1 cells cultured in media with low glucose (4.5 mM) in a dose-dependent manner (Fig. 2A). To support these findings, we consistently observed that Imatinib treatment significantly increased insulin mRNA levels in resting NIT-1 cells (Fig. 2B). Furthermore, knockdown of *c-Abl* using siRNA led to approximately 70% reduction of c-Abl mRNA by real time RT-PCR (data not depicted), and enhanced insulin production by NIT-1 β cells (Fig. 2C). Furthermore, when *c-Abl* siRNA transfected NIT-1 cells were treated with Imatinib, insulin production could be further enhanced (Fig. 2C). The above findings strongly indicate that c-Abl is a negative regulator that controls insulin expression in β cells under resting state when insulin is not needed.

### Glucose Stimulation of NIT-1 β Cells Up-regulates Both c-Abl and Insulin Expression

**Figure 3:**
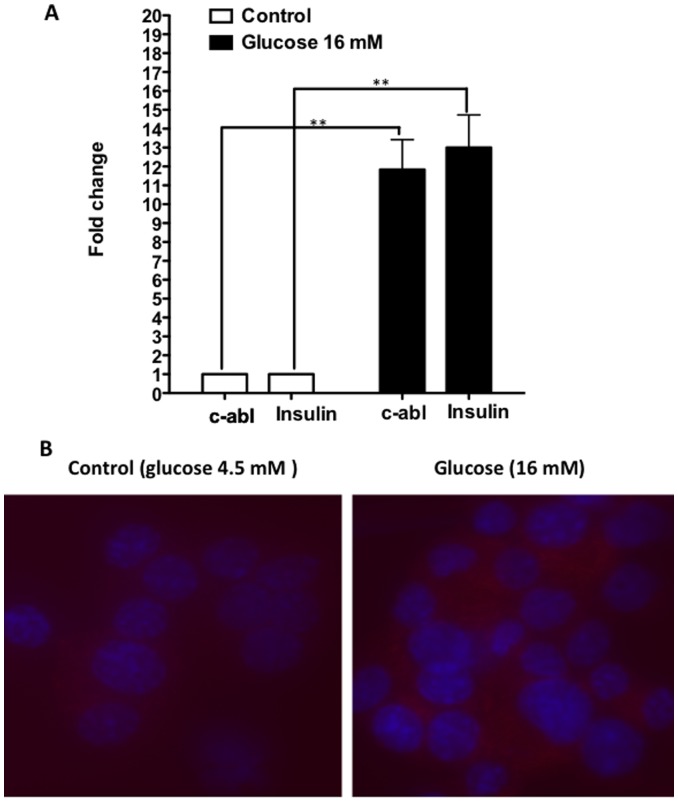
Glucose stimulation induces up-regulation of both c-Abl and insulin expression. **A** . NIT-1 cells were cultured in low glucose DMEM medium with or without glucose 16 mM for 6 hrs. Thereafter, the cells were examined for c-Abl and insulin gene expression using real-time RT-PCR. Student t test was performed. The results shown were from a representative of 3 independent experiments. *: p>0.05, **:p<0.001. **B.** NIT-1 cells were cultured in low glucose DMEM medium with or without glucose 16 mM for 24 hrs. Then, the cells were cytospun onto slides and stained with anti-c-Abl antibodies and Dappi, and visualized by a fluorescent microscope (Zeiss Axioskop). A representative image of three slides in each group is shown.

As shown above, the inhibition of c-Abl up-regulated insulin production induced by glucose, it is of interest to investigate how glucose stimulation influences c-Abl gene expression. We postulated that glucose stimulation would suppress c-Abl, the inhibitor of insulin gene expression so that insulin production could be enhanced. To our surprise, glucose stimulation significantly enhanced both c-Abl and insulin expression (Fig. 3A and B). This result implicates that glucose induces up-regulation of insulin and c-Abl probably via two different pathways, and it is likely that c-Abl up-regulated by glucose stimulation in turn acts on certain elements in the insulin production pathway to regulate and maintain insulin expression at proper levels.

### C-Abl Attenuates Insulin Expression without Involvement of PDX-1

**Figure 4:**
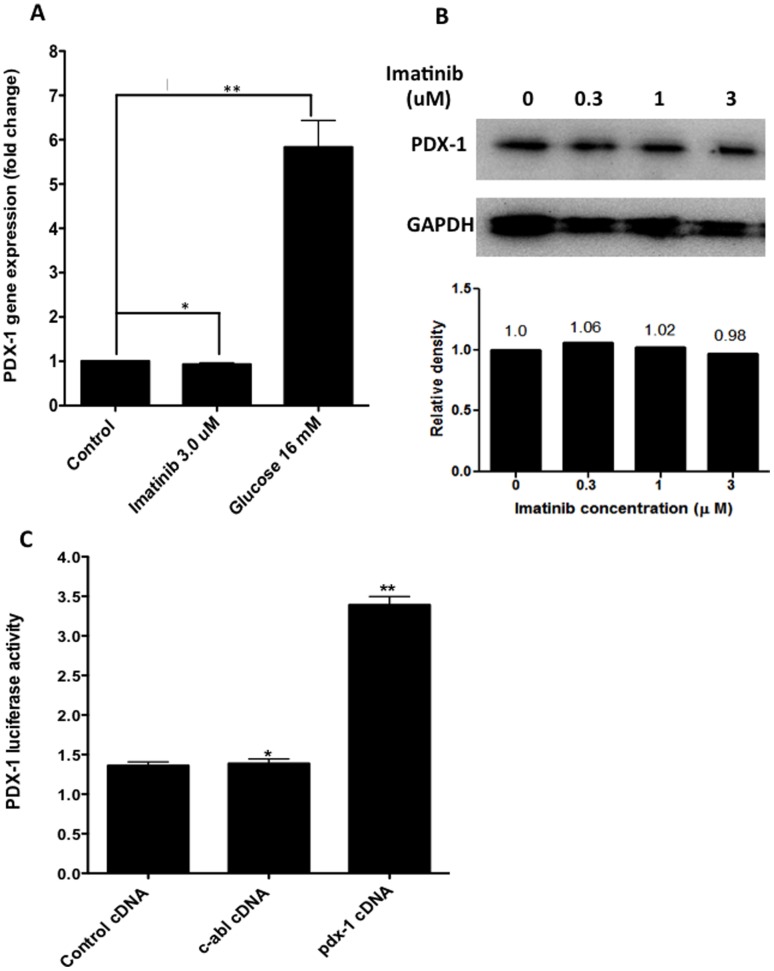
Relationship between c-Abl and insulin gene transcription factor PDX-1. **A**. NIT-1 cells were cultured in the low glucose DMEM medium, or with glucose (16 mM) or with Imatinib (3 µM) for 6 hrs. The cells in each group were harvested and the expression of PDX-1 was examined by real-time RT-PCR. The results shown were from a representative of 3 independent experiments. **B**. NIT-1 cells were cultured in the low glucose DMEM medium with different concentrations of Imatinib (0, 0.3, 1, 3 µM) for 24 hrs. Then, the protein levels of PDX-1 and GAPDH were examined by Western blot. Relative quantity of each PDX-1 band relative to its GAPDH control was shown below the Westerblot image. These results were reproduced by additional two experiments. **C**. 293 cell line were transfected with pdx-1 promoter-driven luciferase reporter gene, together with transfection of plasmids encoding *c-Abl* gene or control plasmids. Twenty four hrs later, the cells from the above conditions were harvested and luciferase activities were measured by using the Dual Luciferase Reporter Kit. This experiment was repeated twice with similar results. The targeted gene expression levels were normalized relative to β actin. One-way ANOVA with post hoc test was performed. *: p>0.05, **:p<0.001.

PDX-1 is a major insulin transcription factor in β cells by binding to A boxes (AT-rich elements), upstream of the insulin gene to enhance gene expression [12], [13]. To determine whether increased insulin production in Imatinib-treated β cells is associated with PDX-1, we studied PDX-1 mRNA levels by real-time PCR in NIT-1 cells under different conditions. Surprisingly, we failed to observe any influence of Imatinib on PDX-1 transcriptional levels (Fig. 4A). To confirm that c-Abl inhibition by Imatinib does not affect PDX-1 expression, we performed experiments for the protein levels of PDX-1 expressed by NIT-1 cells treated by different concentrations of Imatinib using Western blot. Consistent with the results shown in Fig. 4A, Imatinib did not affect PDX-1 protein expression (Fig. 4B).

To further confirm that PDX-1 is not involved in c-Abl-regulated insulin gene expression, we then overexpressed c-Abl by transfecting a *pdx-1* promoter-driven luciferase reporter gene into 293 cell line along with control plasmid or *c-Abl* plasmid or positive *pdx-1* plasmid. As shown in Fig. 4C, *c-Abl* transfection did not affect *pdx-1*-promoter-driven luciferase activity compared to control. While these data would not be able to rule out the regulatory effect of c-Abl on PDX-1 gene expression in real β cells because 293 cell is not β cell-derived cell line, our findings at least indicate that c-Abl does not directly interact with transcriptional regulatory elements of PDX-1 gene. Consistent with the previous report showing that PDX-1 serves as a positive regulator for the gene expression of its own [14], we found that transfection of *pdx-1* plasmid markedly induced PDX-1-driven luciferase activity (Fig. 4C).

### C-Abl Regulates Insulin Gene Expression in β Cells Likely via Regulating NKx2.2

**Figure 5:**
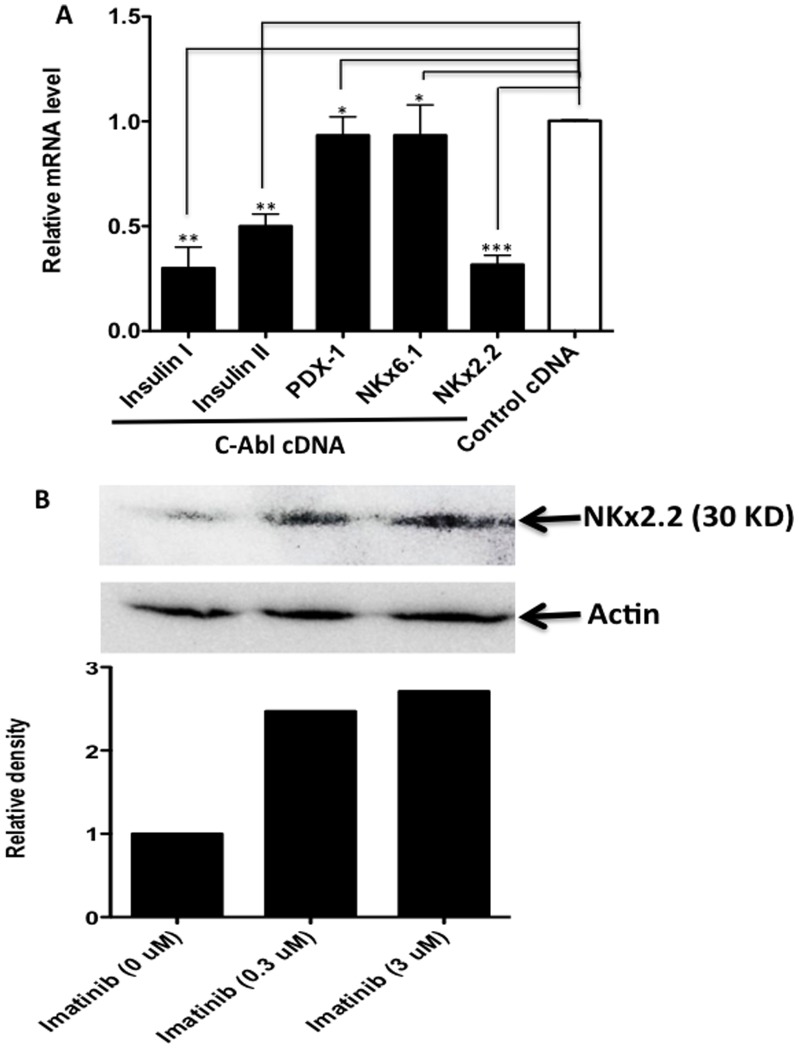
C-Abl regulates insulin gene expression via NKx2.2. **A**. NIT-1 cells were tranduced with retrovirus vector encoding c-Abl gene to overexpress c-Abl or control cDNA. Twenty-four hrs later, the cells were examined for the gene expression of insulin, PDX-1, NKx6.1, NKx2.2, respectively by real-time RT-PCR. The PCR results were normalized relative to β actin for each individual gene, and the relative values of c-Abl transduced cells were compared to those of control cDNA transduced cells which were defined as 1 (the white bar). The results shown were from a representative of 3 independent experiments. One-way ANOVA with post hoc test was performed. *: p>0.05, **:p<0.01, ***:p<0.001. **B**. NIT-1 cells were cultured in the low glucose DMEM medium alone (Immatinib 0 µM), or in the presence of 0.3 µM Imatinib, or 3 µM Imatinib (3 µM) for 24 hrs. The protein levels of NKx2.2 were examined by Western blot. The densitometry was analyzed relative to the levels of β-actin, and the relative level of the cells incubated with 0 µM Imatinib was defined as 1.

To further establish the role of C-Abl in regulating β cell insulin gene expression, we used NIT-1 cells to create a β cell line that overexpresses c-Abl by transfection of a *c-Abl*-retrovirus, and then studied the changes of insulin gene expression and the expression of insulin-related genes. As shown in Fig. 5, we demonstrated that the overexpression of c-Abl in β cells significantly suppressed β cell insulin gene expression. In line with the data presented in Fig. 4, c-Abl overexpression did not affect PDX-1 gene expression and its downstream gene of NKx6.1 (Fig. 5A). We observed, however, that another insulin gene expression regulatory factor, NKx2.2 was dramatically reduced in β cells with c-Abl overexpression (Fig. 5A). The NKx2.2 is a strong activator of NeuroD1 gene, which controls insulin gene expression in response to glucose stimulation [15]. Mouse insulin has two forms (insulin I and II) expressed by two different insulin genes located in chromosome 19 and 7, respectively [16], so we examined the expression levels of both insulin genes. Consistent with the findings described above, we found that the gene expression levels of both insulin I and II were dramatically decreased in c-Abl overexpressed NIT-1 cells in contrast to control cDNA-transduced cells (Fig. 5A). The above data indicate that c-Abl suppresses insulin gene expression likely through NKx2.2 without the involvement of PDX-1. To support NKx2.2 is indeed involved in Imatinib treatment-induced insulin up-regulation, we further demonstrated that c-Abl inhibition by Imatinib markedly enhanced NKx2.2 protein levels in NIT-1 cells (Fig. 5B). Consistent with the above findings, we also found that Imatinib treatment markedly up-regulated gene expression of NeuroD1 (supplemental Fig. S1), which is the down-stream of, and positively regulated by NKx2.2.

### C-Abl Inhibition by Imatinib Promotes GLUT2 Expression on β Cells

**Figure 6:**
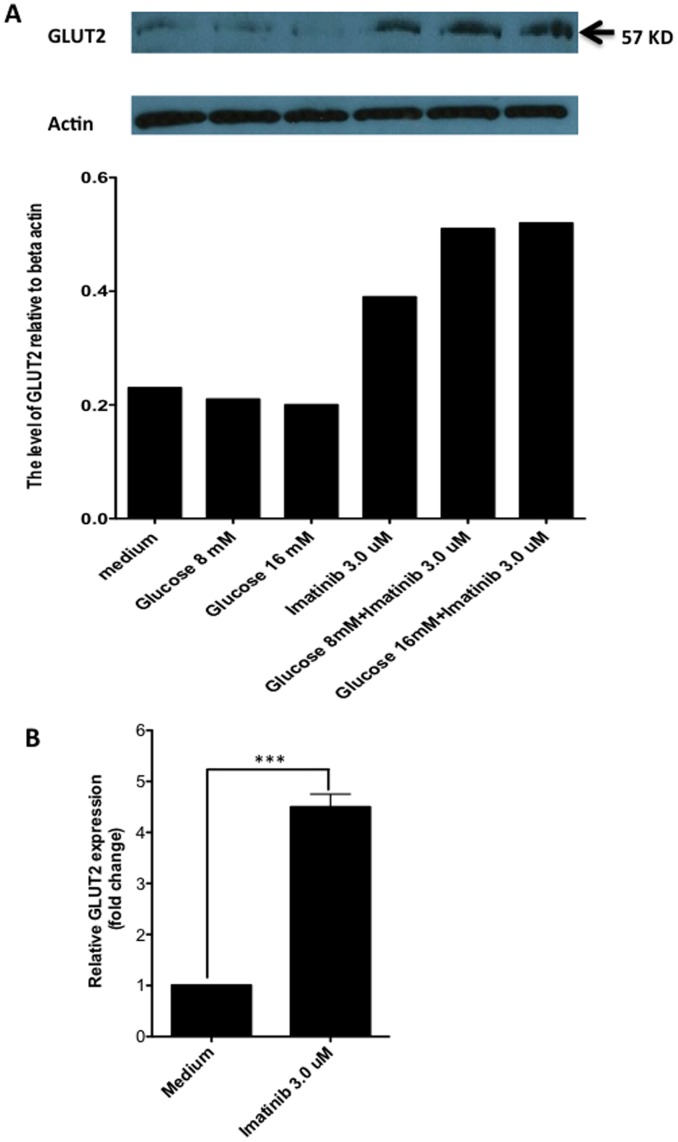
c-Abl inhibition up-reguates GLUT2 expression on β cells. **A**. NIT-1 cells were cultured in low glucose DMEM alone or with 8 mM glucose, 16 mM glucose, 3 µM Imatinib, 8 mM glucose+3 µM Imatinib, or 16 mM glucose+3 µM Imatinib for 24 hrs. The GLUT2 protein levels were evaluated using Western blot. The expression levels were calculated relative to β actin. The similar results were obtained in additional two independent experiments. **B**. NIT-1 cells cultured in low glucose DMEM media alone, or with Imatinib 3 µM for 6 hours. The cultures were triplicated for each condition. Then the cells were harvested and RNA was extracted. The GLUT2 mRNA levels were examined by Real-time RT-PCR. Student t test was performed. ***:p<0.001. The data shown were from one representative of three independent experiments.

It is well known that glucose is a strong stimulator for β cells to produce insulin. Thus, the increase of glucose concentration in cytosol of β cells would stimulate insulin expression. To determine whether glucose intracellular transportation participates in the Imatinib-induced insulin expression, we treated NIT-1 cells with Imatinib and then GLUT2 mRNA and protein levels were evaluated by real-time RT-PCR and western blot, respectively. We found that c-Abl inhibition by Imatinib significantly enhanced GLUT2 expression at both protein and mRNA levels (Fig. 6A and B), suggesting that GLUT2 may participate in up-regulation of insulin induced by Imatinib.

## Discussion

Imatinib was first used in the treatment of chronic myelogenous leukemia (CML) to inhibit c-Abl tyrosine kinase which is constantly activated because of the fusion protein encoded by bcr-abl fusion gene due to chromosome 22 and 9 translocation [17]. Later, it was also used for other diseases with enhanced c-Abl activity, such as GIST [18]. Recently, it was evaluated in both type 1 and type 2 diabetes for its potential application in diabetes management because it was noted that CML patients with diabetes who took Imatinib to control CML also showed improvement in their diabetes [5], [19]. It has demonstrated that CML patients with type 2 diabetes have markedly elevated levels of adiponectin, implicating a mechanism for improved insulin sensitivity in the peripheral tissues [9], [20]. Research in streptozotocin-induced diabetes or type 1 diabetes mouse models has demonstrated that Imatinib prevents β cell apoptosis [8], [21] and islet inflammation [7]. Recently, Mokhtari et al reported that Imatinib improved survival of insulin-producing β cells via inducing phosphatidylinositol 3-kinase signaling [22]. However, there has been no report on whether Imatinib directly acts on β cells to promote insulin production. It could be possible that Imatinib promotes β cell insulin secretion leading to amelioration of diabetes. Supporting this idea, clinical observations demonstrate that CML patients with type 2 diabetes have enhanced levels of serum C-peptide while taking c-Abl tyrosine inhibitor [23]. It is also likely that Imatinib treatment improves diabetes through different mechanisms working together, e.g. providing β cell survival signals and at the same time promoting insulin production.

In the present study, we report a novel mechanism for β cells to regulate their insulin expression. To our knowledge, this is the first to demonstrate that c-Abl tyrosine kinase, as a negative regulator, controls insulin expression in β cells. Tremendous volume of work has been done to elucidate the mechanisms that stimulate insulin production, however, the mechanisms that negatively regulate insulin expression, especially when β cells are under resting state, have drawn much less attention. In fact, positive and negative regulators for β cell insulin production are equally important and both have to be physiologically active and working in concert to control insulin production within a normal range. Through studying the effect of c-Abl tyrosine kinase inhibitor, Imatinib on β cell insulin production, we reveal that c-Abl is an important negative regulator for β cell insulin expression. This regulatory mechanism functions not only in glucose-stimulated β cells but also in β cells at resting state.

The detailed mechanisms of how c-Abl negatively regulates insulin expression are still not clear. However, we have ruled out the possibility that c-Abl down-regulates the expression of PDX-1 which is the major factor promoting insulin production by showing that inhibition of c-Abl using Imatinib does not alter the levels of PDX-1 expression. To support this, we further show that c-Abl tyrosine kinase does not directly affects *pdx-1* promoter-driven luciferase reporter gene expression in a gene transfection cell model using the 293 cell line. To further determine what factors are being involved in down-regulation of insulin by c-Abl, we investigated NKx2.2, which regulates insulin-gene expression-associated transcriptional factor, neuroD1 [15], [24]. We found that overexpression of c-Abl in β cells reduced gene expression for both insulin and NKx2.2 to similar degrees. In line with the finding that c-Abl activity had no relationship with PDX-1, we did not observe any change in the expression of PDX-1 associated transcription factor, NKx6.1 [25]–[28]. Our western blot results provide further support of c-Abl’s negative regulatory role for NKx2.2 because inhibition of c-Abl by Imatinib markedly enhances NKx2.2 levels.

**Figure 7:**
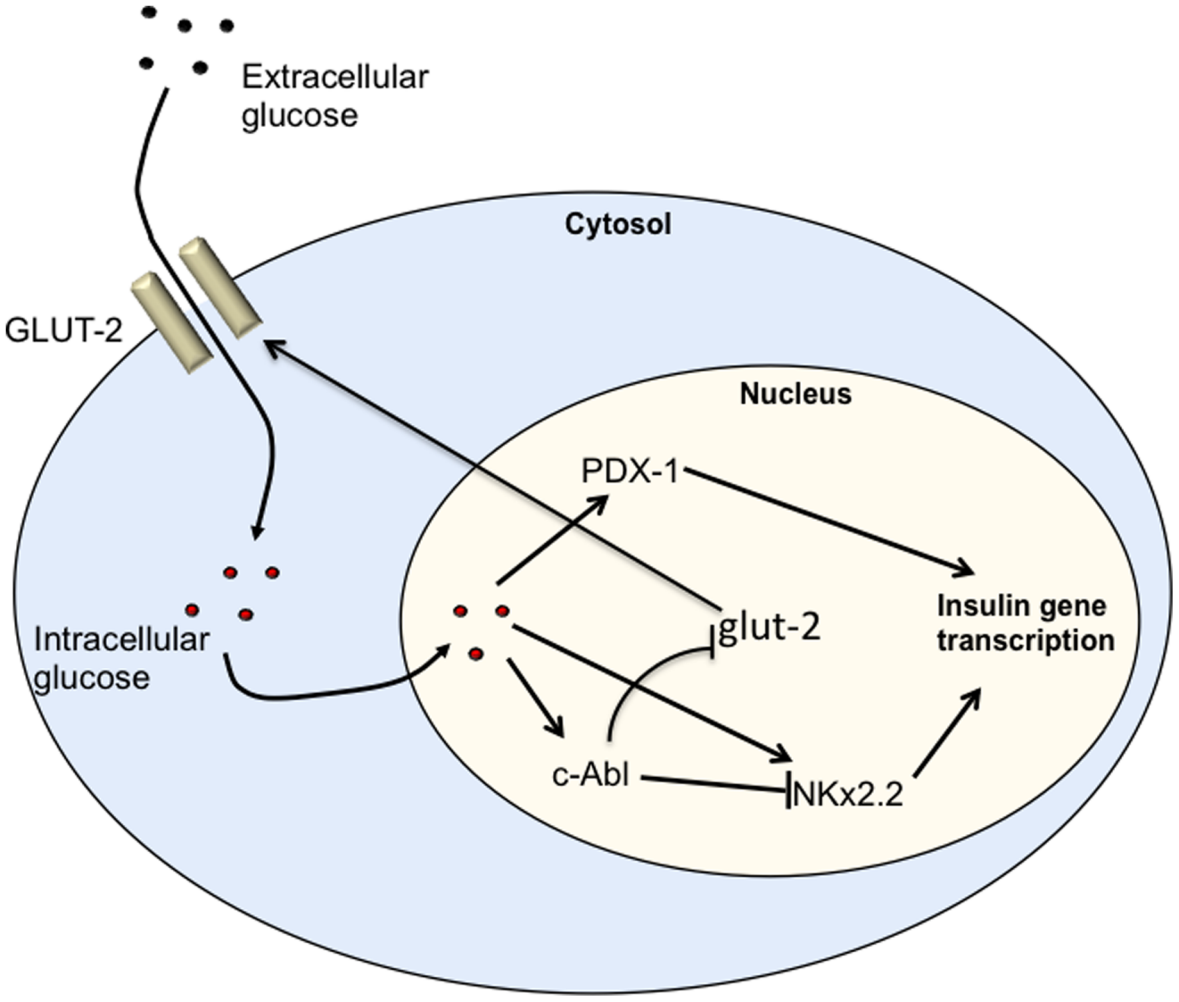
Diagram of c-Abl regulating insulin gene expression. β cells receiving glucose signals upon glucose intracellular transportation by GLUT2 transporters up-regulate insulin gene positive regulatory factors, e.g. PDX-1 and NKx2.2 to enhance insulin gene expression. On the other hand, β cells initiate the negative regulatory pathway through up-regulating C-ABL expression, and C-ABL in turn down-regulates gene expression of NKx2.2 and its downstream insulin gene, as well as GLUT2.

It is known that glucose is the most important stimulator for insulin secretion physiologically. Our data show that inhibition of c-Abl by Imatinib works in synergy with glucose stimulation to promote insulin production. It is of interest to know how glucose stimulation affects c-Abl expression. Given the data presented above, we postulated that glucose would suppress this insulin negative regulator to promote insulin production. Surprisingly, we observed that glucose stimulation dramatically enhanced the expression of c-Abl in β cells. This finding reveals an important regulatory mechanism for insulin secretion under high-level glucose conditions, where glucose not only activates insulin positive regulators to increase insulin production, but also induces negative regulators such as c-Abl to prevent overexpression of insulin, so that an appropriate level of insulin can be maintained. Thus, c-Abl may serve as an important gatekeeper for β cell insulin expression and secretion. The second level of regulating insulin expression by c-Abl may be associated with affecting glucose transporter GLUT2 on β cells. Indeed, we observed that c-Abl inhibition by Imatinib considerably promoted GLUT2 expression on β cells, which would enhance intracellular glucose transportation and then stimulated insulin expression. As shown in Figure 7, we propose the following pathway that in response to high-level glucose, the glucose transporter GLUT-2 on β cells first transports glucose molecules inside the cell, whereupon glucose then activates insulin gene transcriptional regulators, such as PDX-1, NeuroD1 and MafA [29]–[31] to up-regulate insulin expression. One the other hand, glucose also activates c-Abl gene expression as negative feedback to control insulin gene expression and to limit levels of glucose transporter GLUT-2. The timing of glucose initiating these two opposing processes is still unclear. Both could be initiated simultaneously or sequentially. It is likely that glucose and c-Abl affect insulin expression independently but cooperatively. Another important point of this study is that c-Abl likely remains active to control insulin gene expression in resting β cells when insulin is not needed.

The molecular interactions among the molecules associated with regulating insulin gene expression need to be further investigated, such as how NKx2.2 expression is regulated by c-Abl and how c-Abl inhibition by Imatinib up-regulates GLUT2 on β cells. Importantly, animal studies will also be needed to study how c-Abl inhibition by Imatinib affects β cell function in vivo. These issues as well as the results demonstrated in the current study are of importance in guiding the development of new therapeutic approaches for type 1 and type 2 diabetes. Given the discovery of cell permeable small-molecule c-Abl tyrosine kinase activator [32], insulinoma can be potentially managed by using c-Abl activator to inhibit insulin production.

## Supporting Information

Figure S1 **The effect of c-Abl inhibitor Imatinib on NeuroD1 expression.** NIT-1 cells were treated incubated at low glucose medium and medium with 16 mM glucose, in the presence of different concentrations of Imatinib shown in the above figure for 6h, then the cells were harvest and the expression of NeuroD1 were examined by real-time PCR. The results demonstrated that Imatinib significantly promoted NeuroD1 expression.
(TIFF)
