# Supplementary figures and images for: C-Abl Inhibitor Imatinib Enhances Insulin Production by β Cells: C-Abl Negatively Regulates Insulin Production via Interfering with the Expression of NKx2.2 and GLUT-2
Source: PLoS One. 2014 May 16;9(5):e97694. doi: 10.1371/journal.pone.0097694 (PMC4023982; doi:10.1371/journal.pone.0097694)

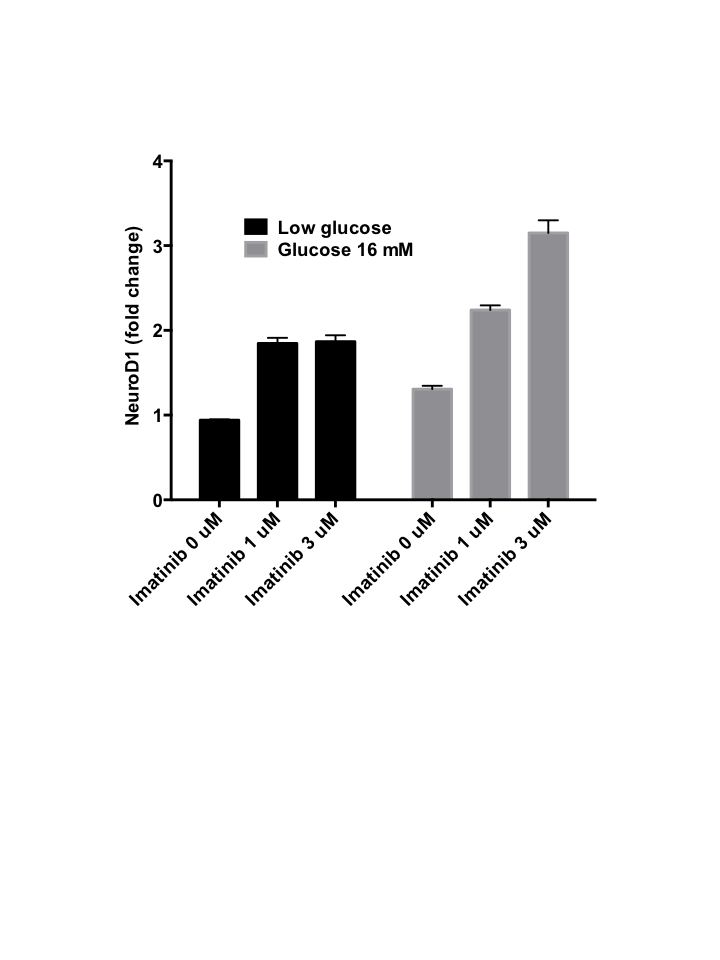

Supplement: Figure S1 — The effect of c-Abl inhibitor Imatinib on NeuroD1 expression. NIT-1 cells were treated incubated at low glucose medium and medium with 16 mM glucose, in the presence of different concentrations of Imatinib shown in the above figure for 6h, then the cells were harvest and the expression of NeuroD1 were examined by real-time PCR. The results demonstrated that Imatinib significantly promoted NeuroD1 expression. (TIFF) [file pone.0097694.s001.tiff]
